# Optical Properties of Biomass Burning Aerosols from Simulated Wildfires and Prescribed Fires with Representative Fuel Beds from the Southeast United States

**DOI:** 10.1021/acsestair.4c00091

**Published:** 2024-08-10

**Authors:** Zachary C. McQueen, Ryan P. Poland, Chase K. Glenn, Omar El Hajj, Robert Penland, Anita Anosike, Kruthika V. Kumar, Joseph J. O’Brien, Rawad Saleh, Geoffrey D. Smith

**Affiliations:** † Department of Chemistry, University of Georgia, Athens, Georgia 30602, United States; ‡ School of Environmental, Civil, Agricultural and Mechanical Engineering, University of Georgia, Athens, Georgia 30602, United States; § U.S. Department of Agriculture Forest Service, Southern Research Station, Athens Prescribed Fire Science Laboratory, Athens, Georgia 30602, United States

**Keywords:** Optical properties, smoke, wildfires, prescribed fires, photoacoustic, absorption, biomass burning

## Abstract

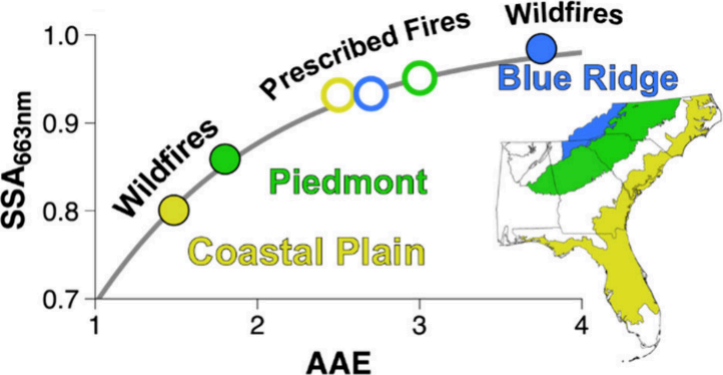

We report measurements of the absorption Ångström exponent (AAE) and single scattering albedo (SSA) of biomass burning aerosol from the combustion of fuel beds representing three eco-regions of the Southeast U.S. (Piedmont, Coastal Plain, and Blue Ridge Mountains) with moisture content representative of wildfires and prescribed fires. We find a strong correlation between the AAE and SSA for both simulated wildfires (low fuel moisture) and prescribed fires (higher fuel moisture). For wildfires, the AAE and SSA are strongly dependent on the eco-region of the fuel bed and span a much wider range (AAE = 1.3–4.2, SSA = 0.75–0.97) than they do for prescribed fires (AAE = 2.4–3.1, SSA = 0.88–0.96). The AAE and SSA are also found to be correlated with the fraction of total carbon that is elemental carbon (*f*_EC_) for both wildfires and prescribed fires, but the range of *f*_EC_ observed (0.02–0.14) from the fuel beds is much smaller than that reported previously from laboratory studies using individual fuels. The observations from the present study suggest that fuel-bed composition and moisture content are significant factors in determining the relative amount of organic material in biomass burning aerosols and, consequentially, their optical properties.

## Abstract 2

This work investigates how fuel-bed composition and moisture content influence combustion conditions and the corresponding optical properties of the biomass burning aerosols generated.

## 1 Introduction

Wildland fires, originating from both wildfires and prescribed fires, are significant sources of biomass burning aerosols (BBAs) globally, and can potentially pose threats to wildlife and human health.^1,2^ However, despite these concerns, wildland fire continues to be a vital ecological process that can enrich soil, help maintain ecosystems, and restore habitats.^3−5^ Wildfires can become destructive because of the potential speed with which they can spread and their high intensities due to low fuel moisture content.^6^ Prescribed fires, on the other hand, are a common forest management tool that can be used to reduce fuels available to wildfires and control the spread of invasive species and are usually ignited when fuel moisture content is higher.^6−8^ BBAs emitted from both types of wildland fires frequently degrade regional air quality and impact tropospheric chemistry and radiative transfer in the atmosphere.^1,9,10^ The direct radiative impacts of BBAs, through absorption and scattering of incoming solar radiation, represent significant uncertainties in climate models.^11−17^

With the frequency and intensity of wildfires, which occur when fuel moisture content is low, increasing due to climate change,^3^ mitigation techniques such as prescribed fires are becoming increasingly important. Despite the growing need for prescribed fires, more effort has been dedicated to studying wildfires and their suppression than understanding the dynamics and emissions from prescribed fires.^18^ In most instances, the primary difference between wild and prescribed fires is in the moisture content of the fuel.^19^ Dry fuel beds in wildfires promote intense flaming fires that spread quickly and consume large amounts of fuel. Conversely, prescribed fires, with higher moisture content, typically burn with lower intensity so that the fire does not propagate as quickly and specific fuels are unavailable for combustion due to high moisture content.^19^ In the Southeast United States, prescribed fires burn a much greater area than do wildfires, and they emit high particulate matter concentrations more often than do wildfires.^9^ Wildfires still occur, however, especially following extended periods of drought, as exemplified by the 2016 wildfire outbreaks in the Southern Appalachian Mountains.^6,20^ In the case of wildfires occurring in eco-regions containing significant duff layers, aerosol emission factors are much higher due to the prolonged smoldering of the duff layer.^19,21^ Direct measurements of the optical properties of BBA emissions from both wild and prescribed fires under controlled experimental conditions will improve the understanding of how they differ.

The chemical composition of BBAs from wildland fires is complex, but two primary components contribute to the absorption of visible solar radiation: “black carbon” (BC) and “brown carbon” (BrC). Black carbon, a potent absorber of light across the visible spectrum with weak wavelength dependence, is characterized by an absorption Ångström exponent (AAE) near 1 and a single scattering albedo (SSA) ≪1.^22−24^ Organic aerosol components that absorb light constitute BrC, which is distinguishable from BC because it typically has a stronger wavelength dependence (AAE > 2) and much higher single scattering albedo (SSA > 0.95).^25−30^

There have been many field campaigns and laboratory experiments to study the optical properties of BBAs emitted by wildland fires.^31−40^ These optical properties have been found to be strongly dependent on the types of fuels burned and to be correlated with the amount of organic aerosol emitted relative to BC.^32,34^ Several field studies have measured BBA optical properties from wildfire events;^31,37^ however, there have been fewer such studies of prescribed fire emissions,^35^ and systematic comparisons of the optical properties of BBA emissions from wildfires and prescribed fires are scarce.^1,31,37^ The study of Marsavin et al.^41^ is a notable exception, as they measured the optical properties of both wildfire and prescribed fire BBAs in the Pacific Northwest of the United States and concluded that BBAs from prescribed fires exhibit absorption properties that are more similar to BC than do BBAs from wildfires. Nonetheless, additional systematic studies of the optical properties of emitted BBAs from these two types of wildland fires are needed.

In the present study, we compare the measured AAE and SSA of BBAs emitted from simulated wildfires and prescribed fires with fuel beds representative of three different eco-regions of the Southeast United States. We also measure the ratio of elemental carbon to total carbon content to provide insight into how fuel moisture content changes the composition of the emitted BBAs and explore how the aerosol optical properties are correlated with this composition. This work helps to bridge the gap between laboratory and field measurements of BBAs by demonstrating how the use of heterogeneous fuel beds, rather than individual fuels, leads to BBA optical properties that are more representative of those found from wildland fires in their natural landscape context.

## 2 Methods

### 2.1 Fuel Collection and Fuel Bed Construction

All measurements in this study were made as part of the Georgia Wildland Fire Simulation Experiment (G-WISE) at the U.S. Forest Service Prescribed Fire Science Laboratory (U.S. Forest Service Southern Research Station, Athens, GA, United States) from October 25, 2022, to November 19, 2022. Fuels were collected from the Oconee National Forest, Fort Stewart Military Reservation, and the Chattahoochee National Forest representing the Piedmont (P), Coastal Plain (CP), and Blue Ridge Mountains (BR) eco-regions, respectively. Figure S2 displays a map of the eco-regions studied here as well as the approximate locations for the sampling sites. Individual fuels were separated into four types: pine needle, woody fuels, surface litter (leaves, pinecones, etc.), and duff. Woody fuels were cut to 20 cm in length and categorized by diameter size class to represent fuel moisture time lags (1, 10, and 100 h).^42^ Fuel moisture content upon retrieval was measured using a Model MAX 4000XL - Moisture & Solids Analyzer (Computrac), and then the fuels were dried in an oven set to 65 °C for 48 h and re-analyzed for moisture content. All wildfire-conditioned fuel beds were left at the moisture content following drying, and each fuel was weighed. Prescribed fire fuels were humidified differently based on the fuel. Woody fuels were saturated by submerging in water and then dried to the desired moisture content. Fine fuels were placed in a walk-in humidifier until the desired moisture content was reached. Duff samples for prescribed fire simulations were left at the high moisture content at which they were collected (50–60%).

Fuel beds were constructed based on the mass percentage of individual dry fuel types found in each eco-region (Table 1). P and CP fuel beds were dominated by dead pine needle (50% and 56%, respectively), while BR fuel beds were dominated by the duff layer (92%) with deciduous leaf litter and small diameter woody fuels comprising the remaining 8%. Differences in the surface fuels that are not categorized as pine needle or woody fuels (“Other”) come from the distribution of forest types in these two eco-regions. P and BR each have a much larger area of deciduous forest than CP,^43^ which would contribute more broad-leaf litter such as oak leaves.^44^ CP, on the other hand, has more shrubs, herbs, and grasses on the forest floor.

**Table 1:** Dry Fuel Breakdown for Each Eco-Region

Eco-Region	Fuel Type	Mass %	Other Composition
Piedmont (P)	Woody	18%	Leaves from broadleaf species such as oak, hickory, and birch^44^
	Pine Needle	50%	
	Other	32%	
			
Coastal Plain (CP)	Woody	20%	Shrubs, herbs, and grasses^43^
	Pine Needle	56%	
	Other	24%	
			
Blue Ridge (BR)	Woody	3%	Leaves from broadleaf species such as oak, hickory, and ash^44^
	Duff	92%	
	Other	5%	

Prescribed fire fuel beds were constructed to the same dry mass percentage as wildfire fuel beds, with the only difference being the moisture added. Fuel beds were placed in a circle with an area of 0.5 m^2^ on a ceramic fiber board, which was placed on a scale that logged the mass loss of fuel over the duration of the burn. A photo of an example fuel bed is shown in Figure S1 in the Supporting Information. P and CP fuel beds each had a total dry fuel load of 500 g, and BR fuel beds had a non-duff dry surface fuel load of 243 g. Duff in BR fuel beds accounted for 2–3 kg for simulated wildfires and 6–7 kg for simulated prescribed fires, the difference being due to the moisture content.

### 2.2 Fuel Bed Ignition

Each burn was conducted in the burn room, which has a volume of 990 m^3^. Prior to each burn, the burn room was vented with industrial fans to remove residual smoke remaining from the previous burn. The edge of each fuel bed was ignited using a propane torch and allowed to burn to completion. Videos of the fires were taken using a GoPro camera (HERO8) and a thermal imaging IR camera (FLIR A655 SC) to monitor the flaming/smoldering phase and radiant energy release of the fuel beds. Once ignited, the fire was allowed to burn until combustion was deemed complete, dictated by the average temperature measured by the IR camera. Burn duration varied by fuel bed and fuel moisture content, with a typical burn for P and CP wildfires lasting 15–30 min but only 8–10 min for prescribed fires. BR wildfire burns lasted much longer, ranging from 140 to 160 min due to the smoldering of the duff layer, but BR prescribed fires, with duff smoldering inhibited, lasted only 10 min.

### 2.3 Elemental and Organic Carbon Analysis

Filters for organic carbon (OC) and elemental carbon (EC) analysis were collected for 30 min at 5 SLPM (standard liters per minute) following the completion of each burn and stored in sealed Petri dishes in a freezer based on the recommendations of Glenn et al.^45^ Particulate matter was collected on two 47 mm quartz filters (PALL, Tissuquartz 2500) in parallel, with a PTFE filter (0.2 μm, Sterlitech Corporation) placed in series before one of them to be used to correct for contributions from volatile species when making the EC/OC measurements. Punch-outs of each filter (1.5 cm^2^ area) were made to be used in the Model 5L OCEC Analyzer (Sunset Laboratory). The OC and EC components of the filter samples were measured following the NIOSH-870 protocol.^46^ The fraction of elemental carbon relative to the total carbon content (*f*_EC_) was calculated by dividing the EC component by the sum of the OC and EC components:
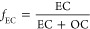
1

### 2.4 Measurement of Particle Optical Properties

BBA absorption coefficients were measured at 406, 532, 663, and 783 nm using a four-wavelength photoacoustic spectrometer (PAS), and the extinction coefficient at 663 nm was measured using a cavity ring-down (CRD) spectrometer.^47^ The PAS calibration procedure is detailed in Fisher and Smith (2018), but briefly, we flow NO_2_ gas through both the photoacoustic cell and CRD spectrometer and calibrate the microphone response normalized to laser power to the absorption due to NO_2_, which is directly measured by the CRD. The optical instruments sampled from the main sampling line at 350 SCCM (standard cubic centimeters per minute) in parallel with other online instruments used in the campaign. The total flow of the online instruments was maintained at a flow rate of 10 SLPM. Measurements were made for the absorption and extinction coefficients at 1 s intervals and then averaged to 2 min. Gas-phase backgrounds were taken prior to each sampling period using a HEPA filter (Pall) in the sampling line.

**Figure 1:**
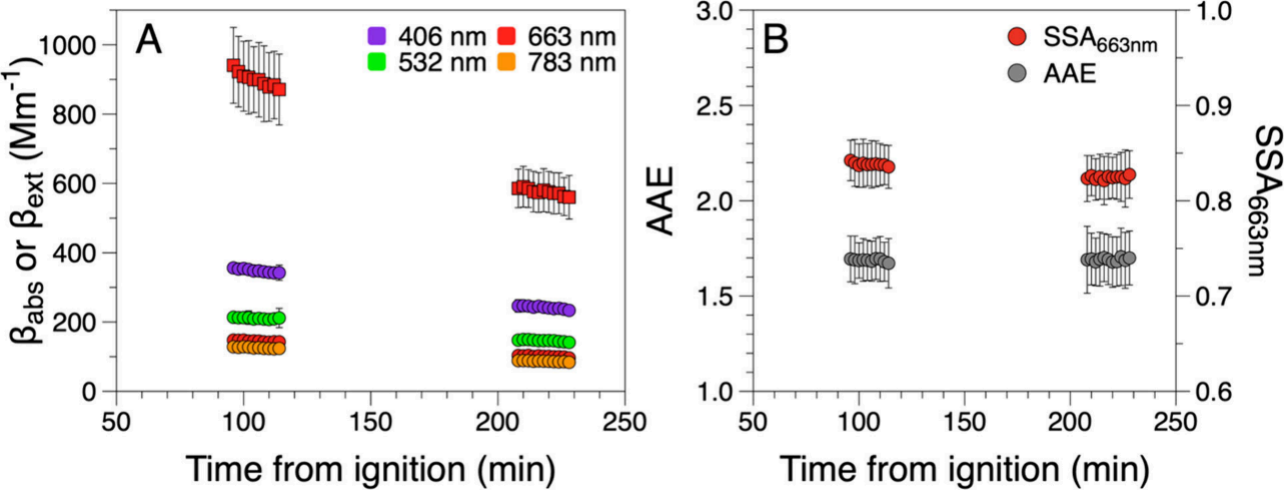
Time-series measurements of the (A) absorption and extinction coefficients and (B) the corresponding AAE and SSA. Data points are 2 min averages of 1 s measurements, and the error bars represent 2 standard deviations of the 1 s measurements. This time series is from a burn of a Piedmont fuel bed with low moisture content carried out on October 25, 2022.

Figure 1A shows an example time series of the absorption and extinction coefficients from a single Piedmont wildfire burn. Figure 1B shows the corresponding time series of the AAE and SSA derived from the absorption and extinction coefficients. The AAE was calculated using all four wavelengths of the PAS by fitting the absorption coefficients to a power law function:
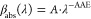
2where β_abs_ is the absorption coefficient at wavelength λ and *A* is a scaling constant. The SSA is the ratio of scattering to extinction and can be calculated directly from the absorption and extinction coefficients at a given wavelength, λ (663 nm in the present work):
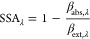
3

The individual 2 min measurements of AAE and SSA were averaged each day to get representative values for each fuel bed.

We also calculated dual-wavelength AAEs from absorption measured at 406 and 532 nm (AAE_BG_), where the absorption is more sensitive to contributions from BrC, and from absorption measured at 663 and 783 nm (AAE_RIR_), where BC is the dominant source of absorption:
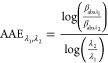
4

## 3 Results and Discussion

### 3.1 The AAE and SSA Are Dependent on Fuel Bed Composition

**Figure 2:**
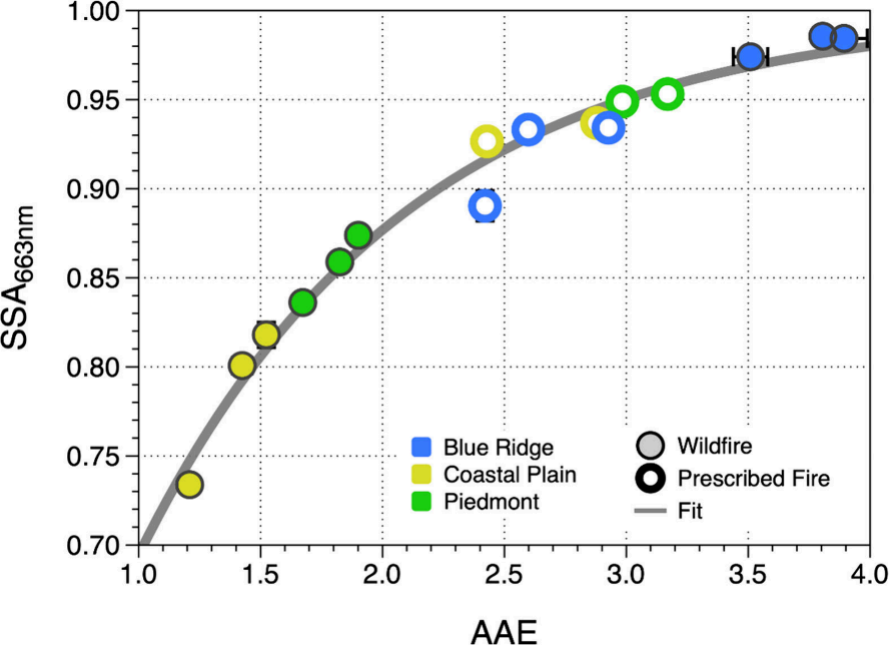
SSA_663 nm_ vs AAE for both wildfires (low fuel moisture content; filled circles) and prescribed fires (higher fuel moisture content; open circles) for the three fuel-bed eco-regions. The gray line represents a fit to the data (SSA_663 nm_ = 1 – 0.76 exp(−0.91 × AAE)) to guide the eye and demonstrates a high degree of correlation (*R*^2^ = 0.98). Error bars represent 2 standard deviations from the representative averages for each day.

We find that there is a high degree of correlation between the AAE and the SSA for all fuel beds and both low and high fuel moisture content from the G-WISE campaign (Figure 2 and Table S1 in the Supporting Information). This correlation is very strong despite differences in the fuel-bed compositions for each eco-region and despite the fact that the burns included both wildfires and prescribed fires. We can interpret the range spanned by these measurements as representing different amounts of BC and BrC contributions, with BC typically possessing AAE values near unity^48^ and BrC typically possessing higher values (>2).^25−30^ Thus, the measurements on the left side of the plot (with AAE near 1) can be considered as being dominated by BC, while the measurements on the right side of the plot (with AAE > 2) as being influenced heavily by BrC. The corresponding SSA values are also a function of the relative amounts of BC and BrC, with BC exhibiting lower values than BrC,^49,50^ and thus they demonstrate a high degree of correlation with AAE in Figure 2.

Figure 2 also highlights the effect of fuel moisture content on the optical properties of the BBAs. For wildfires, the AAE and SSA are strongly dependent on the eco-region, with significant differences between fuel beds that contain only surface fuels (CP and P) and those that contain duff (BR). BBAs from BR fuel beds exhibit large AAE and SSA values indicative of significant BrC contributions to absorption. BBAs from CP and P fuel beds have much lower AAE and SSA values than those from BR fuel beds, indicating that BC is more dominant. Interestingly, the general relationship observed in Figure 2 holds even under the inherent variability of open fires from replicates of a single eco-region/moisture content combination. For example, the three CP wildfire points (filled yellow circles) represent samples from burns on three different days that were conducted under nominally identical conditions, i.e., the same fuel-bed composition and moisture content, yet they demonstrate different AAE and SSA values. This observation suggests that the inherent variability in open fires or from uncontrolled external factors can lead to differences in combustion conditions that result in differences in BBA optical properties. Nonetheless, the observed differences in the optical properties still follow the general trend (gray line in Figure 2).

Measurements of prescribed fires, however, show that the optical properties exhibit a much smaller range (AAE: 2.4–3.2 and SSA: 0.88–0.96) when there is a large amount of moisture present in the fuel beds (open circles in Figure 2). Both the AAE and SSA of the CP and P fuel beds increase relative to measurements made for wildfires, and they overlap with measurements of prescribed fires from BR fuel beds. This increase is representative of aerosols with more BrC character and can be attributed to a change in the combustion conditions from a more intense, flaming fire with wildfires to a less intense, smoldering fire with prescribed fires. The shift to a smoldering-dominated fire consequently leads to an increase in the organic matter emitted from the fire, which directly contributes to the optical properties shifting to a more BrC oriented regime.^51,52^ On the other hand, both AAE and SSA were seen to decrease for BBAs emitted from BR fuel beds combusted under prescribed fire (low fuel moisture content) conditions. We attribute this behavior to the fact that the moisture present prevented the duff layer from combusting as evidenced by the shorter burn times (10 min) compared to those for wildfires (140–160 min). Without the duff burning, the combustion of the surface fuels is the primary contributor to the aerosol emissions, and consequently the BR BBA optical properties are similar to those of the other two eco-regions.

### 3.2 The AAE and SSA Are Correlated with the Relative Amounts of Elemental Carbon and Organic Carbon

**Figure 3:**
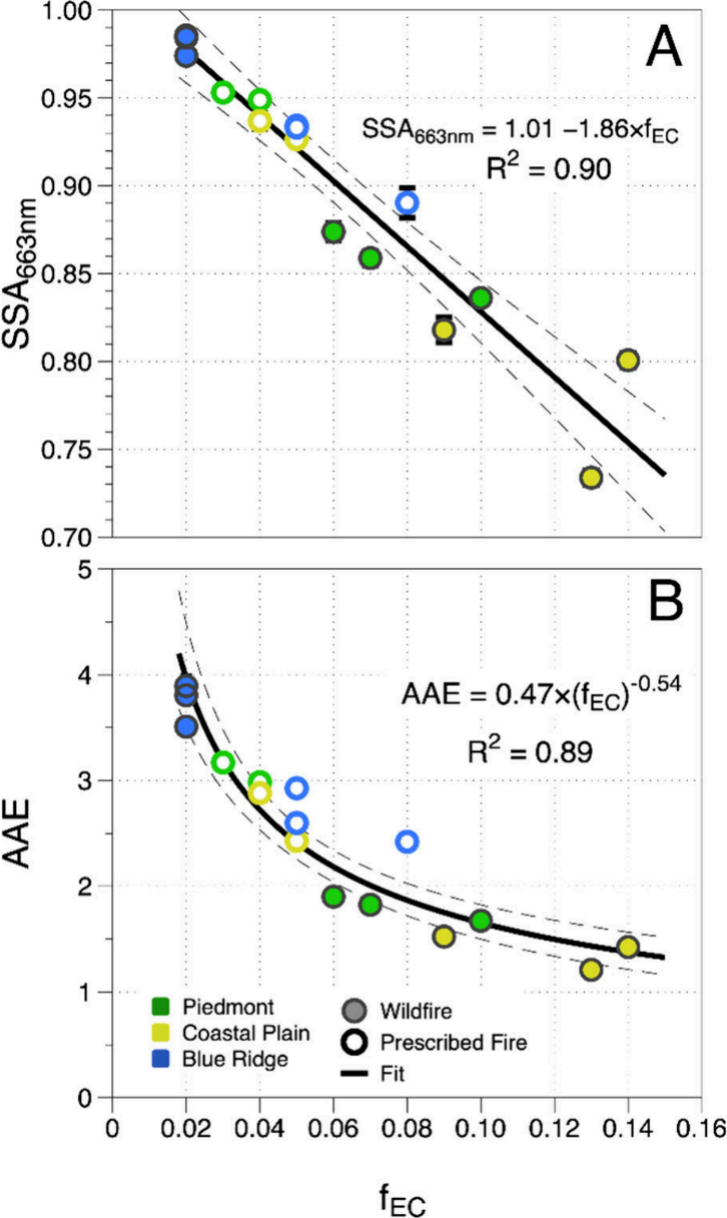
(A) SSA and (B) AAE plotted as a function of *f*_EC_, the fraction of elemental carbon to the total carbon (sum of OC and EC) measured from filter samples. Dashed lines represent the 95% confidence intervals to the linear (SSA) and power law (AAE) fits described by the equations in each panel. Open circles represent prescribed fires and filled circles represent wildfires.

Previous laboratory experiments have demonstrated that BBA optical properties depend on the relative amount of elemental carbon (EC) (or black carbon) and organic carbon (OC).^32,34^ Here, we use the fraction of elemental carbon to the total carbon mass, *f*_EC_ (eq 1), measured using an OCEC analyzer, as a proxy for the composition of the aerosol.^31,33^Figure 3 shows the dependence of the SSA and AAE on *f*_EC_ for the fuel beds of each eco-region for both wildfires and prescribed fires. Figure 3A shows that the SSA has a strong linear correlation with *f*_EC_ (*R*^2^ = 0.90), indicating that this ratio is a useful measure of aerosol composition for predicting SSA even for fuel beds consisting of a mixture of different fuels and for both wildfires and prescribed fires. Figure 3B demonstrates that AAE is also highly correlated with *f*_EC_ (*R*^2^ = 0.89), but it follows a power law functional dependence.

Previous work has used the modified combustion efficiency (MCE) to parameterize the AAE and SSA of BBAs, though it has been demonstrated that they are much more highly correlated with the relative fractions of EC and OC present in the aerosol.^32,34^ We, too, find that the AAE and SSA values are much better correlated with *f*_EC_ (Figure 3) than MCE (Figure S3). However, the slope of SSA_660 nm_ vs *f*_EC_ from the present work (1.86; Figure 3A) is significantly larger than the slope from the work of Pokhrel et al.^33^ (1.07), and the degree of scatter in their plot of AAE vs *f*_EC_ is much larger than what we observe (Figure 3B). These differences suggest that there are other factors influencing the optical properties that are not captured solely by the fractional elemental carbon composition.^33^ Thus, these parameterizations may be useful for specific collections of fuels and/or fuel types, but we caution that they may not be useful in general to predict optical properties solely from measures of the fractional EC composition of BBAs.

Figure 3 also demonstrates that the moisture content of the fuel bed directly impacts the magnitude of the EC fraction of the aerosol and consequently the optical properties. For wildfires, we see that BBAs from P and CP fuel beds have the highest EC fraction (0.06–0.15) and the lowest AAE and SSA values. BR, on the other hand, has a small EC fraction (0.02) and high SSA values (0.97–0.99) with significant wavelength dependence in the absorption (AAE ≥ 3.5), implying that when the organic fraction is large, BrC determines the optical properties.

When fuel moisture content is higher, however, the BBAs from P and CP fuel beds shift to a less EC-dominated region (lower *f*_EC_) due to the lower combustion temperatures and increased smoldering. This shift leads to a more significant contribution from BrC to the optical properties, resulting in higher AAE and SSA values from P and CP prescribed fires. BR prescribed fires, however, show an increase in *f*_EC_ into a range more similar to P and CP fuel beds (0.05–0.08). This increase in *f*_EC_ further supports the conclusion that the duff layer does not contribute to the combustion and that the surface fuels in BR fuel beds produce BBAs with similar optical properties to those of P and CP fuel beds with higher fuel moisture content.

### 3.3 The AAE is Wavelength-Dependent and Varies Based on Fuel Bed Composition and Moisture Content

**Figure 4:**
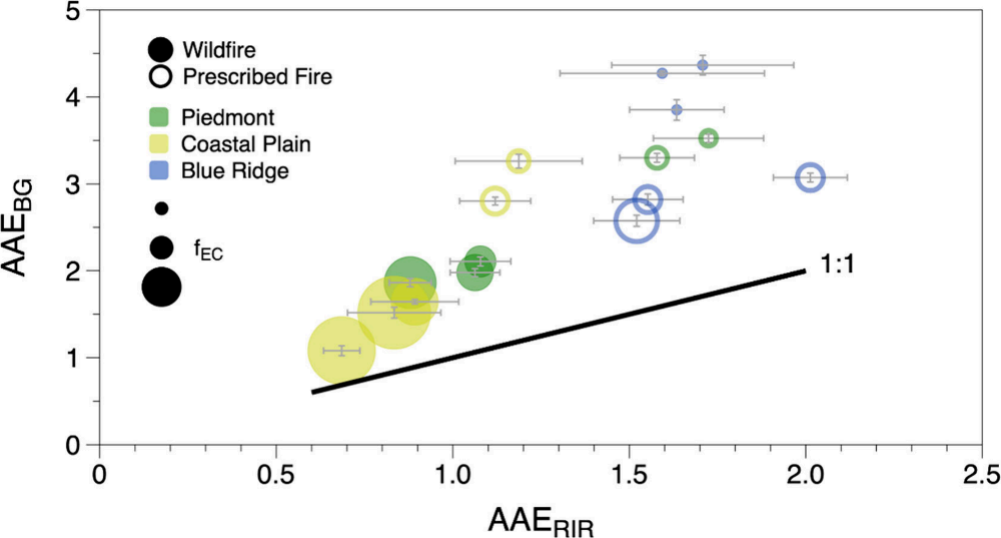
Values of AAE calculated with the 406/532 nm pair of wavelengths (AAE_BG_) and 663/783 nm pair of wavelengths (AAE_RIR_). The error bars, shown in gray, represent 2 standard deviations of the 2 min measurements of each day. Points are color coded by eco-region for both wildfires (filled circles) and prescribed fires (open circles). Size of colored circle is proportional to the fraction of elemental carbon, *f*_EC_, as measured with the OCEC analyzer.

Typically, the wavelength dependence of an aerosol absorption spectrum is quantified by the absorption Ångström exponent (AAE), which is derived from a power law fit to the spectrum (eq 2). Implicit in this approach, however, is the assumption that a single power law function adequately describes the absorption across the entire wavelength range of the spectrum. In fact, we find that the value of AAE depends on the region of the spectrum that is fit, with AAE values calculated with the 406/532 nm wavelength pair (AAE_BG_) systematically larger than AAE values calculated with the 663/783 nm wavelength pair (AAE_RIR_) (Figure 4). Though such a wavelength dependence has been noted previously,^14,53^ it is often overlooked and can have consequences for how the spectral dependence of absorption is interpreted. For example, source apportionment models based on aerosol absorption measurements typically assume that a single AAE value describes a spectrum from a specific source, such as traffic or wood-burning.^54−57^

Having the ability to measure AAE at red and near-IR wavelengths (AAE_RIR_) provides additional insight into how the absorption spectra vary by fuel and moisture content. For example, in Figure 4 we see that AAE_RIR_ ranges from as small as 0.7 to as large as 2.0 with clear trends with fuel-bed composition (color of circle) and moisture content (open/filled circles). The fact that many of these values are different than 1 has significant implications for attempts at separating the BrC and BC contributions; it is common to infer BrC absorption at shorter wavelengths by subtracting a BC spectrum constructed by extrapolating from red/near-IR wavelengths using an assumed AAE = 1.^58^ If the AAE_RIR_ is different from 1, however, then this method could lead to over-/under-estimation of the BrC contributions at shorter wavelengths.

Several of the AAE_RIR_ measurements are significantly greater than 1, which demonstrates that there must be contributions to absorption at the red/near-IR wavelengths other than those from just BC. Specifically, although it is typically assumed that BrC does not absorb appreciably in the red region of the spectrum, it is possible that it does,^59^ thereby giving rise to AAE_RIR_ values greater than 1. Alternatively, clear coatings on BC particles can enhance absorption, which Cappa and Lack,^60^ using Mie theory calculations, found could increase AAE up to 1.6. While we cannot differentiate between these two contributions, it is clear from Figure 4 that either or both of them are present and that their impacts vary greatly according to fuel-bed composition and moisture content.

In general, we find that the values of both AAE_BG_ and AAE_RIR_ tend to correlate inversely with the fraction of elemental carbon, *f*_EC_, which is represented in Figure 4 by the size of the colored circles. Broadly speaking, such a relationship implies that as the amount of organic carbon relative to elemental carbon increases (i.e., *f*_EC_ decreases, the circles become smaller), the role of BrC and/or coating enhancement increases. This trend is logical, as both the amount of BrC and the thickness of coatings on BC particles can be reasonably expected to scale with the amount of organic carbon generated. This trend also helps us to interpret the role of fuel moisture content on the absorption spectra. Looking at the CP and P data points, we see that for wildfires (filled circles), the AAE_RIR_ values are low, ranging from approximately 0.7 to 1.1—consistent with what could be expected for aerosol absorption dominated by BC with little contribution from BrC or coating enhancement. Indeed, the corresponding values of AAE_BG_ are low as well (ranging from 1.1 to 2.2), also indicating absorption dominated by BC. For the same fuel beds burned as prescribed fires (open circles), however, both AAE_BG_ and AAE_RIR_ increase substantially, consistent with the enhanced role of smoldering and the concomitant increase in production of organic carbon that occurs because of the higher fuel moisture content.

For the BR fuel beds, on the other hand, there is little noticeable change in AAE_RIR_ (and AAE_BG_ to a lesser degree) despite the shift from duff-mediated combustion (wildfire) to non-duff fuel combustion (prescribed fire). This is a reasonable observation, though, because in both cases the combustion is dominated by smoldering: promoted by the duff smoldering when fuel moisture content is low (wildfire) and by the surface fuels when fuel moisture content is high (prescribed fire). The smoldering produces more organic carbon, thereby potentially enhancing the roles of both BrC absorption and coating enhancement. Indeed, the P and BR prescribed fire data points (green and blue open circles, respectively) fall in the same range, suggesting similarities in the organic carbon produced from the smoldering of the fuel beds’ surface fuels, each of which contains leaves from broadleaf species.^51^

### 3.4 Aerosols Generated from Combustion of Fuel Beds Exhibit a Narrower Range of Optical Properties Than Do Those from Combustion of Individual Fuels

**Figure 5:**
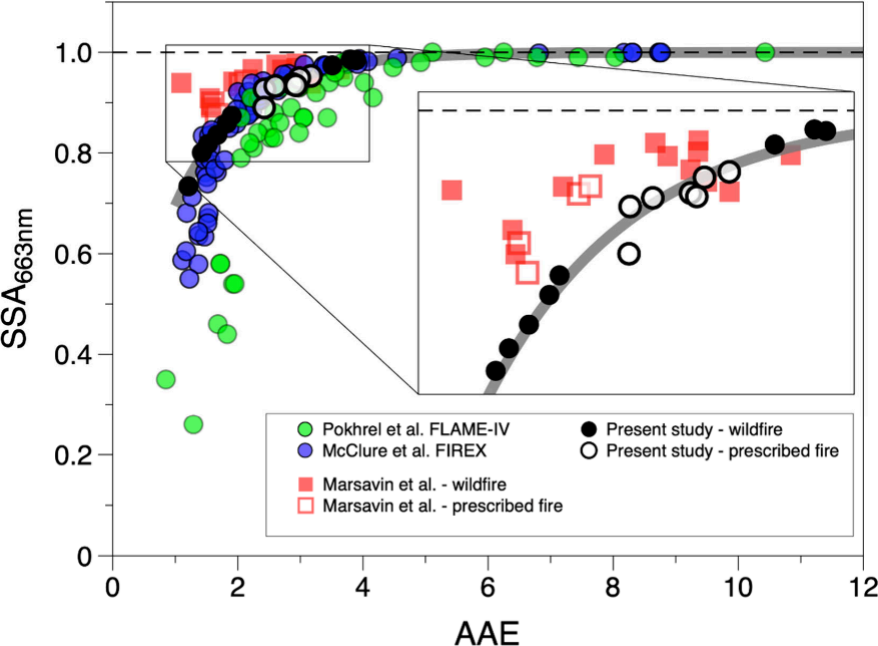
SSA_663 nm_ and AAE from this study (open and closed black circles) compared to previous studies of individual fuels from FLAME-IV^32^ (green circles) and FIREX^34,62^ (blue circles) experiments. Ambient measurements from wildland fires made by Marsavin et al.^41^ in Oregon are also shown (open and closed red squares), and the similarities with the present work are highlighted in the inset. The gray line is the fit to the data from the present study (from Figure 2).

In the present study, fuel beds were constructed to represent those typical of wildland fires in the Southeast United States. As such, the fuels combusted consisted of a mixture of woody fuels, pine needles, leaves, grasses, duff, and other components (see Table 1). Most previous laboratory studies of BBA optical properties, on the other hand, have focused more on burning individual fuels.^31,33,34,61^ In Figure 5, we plot the SSA_663 nm_ and AAE values derived from measurements from two such studies, the FLAME-IV campaign (green circles)^32^ and the FIREX lab study (blue circles).^34,62^ In the present study, we also controlled the fuel-bed moisture content, which was not done in the FLAME-IV and FIREX studies.^63^ Details of how we calculated SSA_663 nm_ from those datasets and the wavelengths used to derive AAE values are given in Table S2 in the Supporting Information. In general, the data points from those studies exhibit the same trend as do the measurements from the present study (open and filled black circles), with the SSA_663 nm_ increasing sharply at low AAE and approaching an asymptote of 1 at high values of AAE. However, in both the FLAME-IV^32^ and FIREX^34^ laboratory studies, duff, peat, and other organic-rich fuels were burned individually, which led to larger values of AAE and SSA_663 nm_ than were observed in the present study. The narrower ranges that we observed for AAE and SSA_663 nm_ can be attributed to the fact that BBAs from the burning of fuel beds are generated from the combustion of a mixture of fuels, which tends to average contributions to the optical properties from individual fuels. This averaging is likely due to the range of combustion conditions with a mixture of fuels being smaller than the extremes of combustion conditions exhibited when burning individual fuels. This is also evident in the range of *f*_EC_ that we measured in this study (0.01–0.17) compared to the FLAME-IV and FIREX studies (0.007–2 and 10^–4^–1, respectively). Consequently, these measured optical properties from fuel beds containing representative fuel mixtures are more likely to represent the optical properties of BBAs from wildland fires than those derived from single-fuel combustion.

Also shown in Figure 5 are the AAE and SSA_663 nm_ values derived from the measurements of Marsavin et al.^41^ at the Mt. Bachelor Observatory in Oregon from wildland fires that occurred in the Cascade Mountains in April to September of 2021 (open and filled red squares). This study provides an interesting comparison with the present study since it is the only one to compare the optical properties of BBAs from both wildfires and prescribed fires. Even though those wildland fires took place in a different eco-region and the primary fuel sources were different (mostly Douglas Fir and other coniferous trees),^41^ the observations from the two studies are similar. For one, the values of SSA_663 nm_ and AAE derived from the measurements of Marsavin et al.^41^ span a much narrower range than do the values derived from the individual-fuel studies, just as we observed from the representative fuel beds in the present study. Also, the values of the optical properties for prescribed fires from the work of Marsavin et al.^41^ span a significantly narrower range than do the values for wildfires, similar to our observations. These similarities confirm that the simulated fires used in the present work generated BBAs that are representative of those from wildland fires. What is more, these similarities suggest that the observed behaviors, namely, the narrowing of the ranges of AAE and SSA_663 nm_ for BBAs from combustion of fuel beds instead of single fuels and from prescribed fires instead of wildfires, may apply to other eco-regions with different fuel-bed compositions.

## 4 Atmospheric Implications

We have shown that the AAE and SSA_663 nm_ of BBAs from fuel beds span a narrower range than those of BBAs emitted from individual fuels, which can influence their impact on radiative transfer in the atmosphere. To explore the potential implications of these measurements for the climate, we use a simple calculation of the radiative forcing efficiency per unit optical depth^64,65^ calculated at 663 nm (RFE_663 nm_):
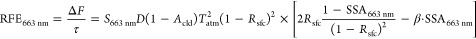
5where Δ*F* is the change in radiative forcing due to the aerosol, τ is the aerosol optical depth, *S*_663 nm_ is the top of atmosphere integrated solar irradiance from 663 to 664 nm from the ASTM-G173-0 solar spectrum (1.585 W m^–2^^66^), *D* is the fractional day length (0.5^67^), *A*_cld_ is the cloud fraction (0.6^67^), *T*_atm_ is the atmospheric transmission (0.79^67^), *R*_sfc_ is the ground surface albedo (0.19^13^), and β is the backscatter ratio (0.17, a typical value for BBAs^50^). A plot of the calculated values of RFE_663 nm_ vs AAE for all of the studies represented in Figure 5 is included in the Supporting Information (Figure S4). While a complete analysis would need to consider the full spectral dependence of the RFE, these calculations specifically at 663 nm nonetheless allow us to assess the relative potential climate impacts of BBAs from the different fuel beds studied.

We find that even over the narrow range of AAE and SSA_663 nm_ values observed in the present study, the RFE_663 nm_ spans a range from −0.021 W/m^2^ (cooling effect) to +0.003 W/m^2^ (warming effect), highlighting the influence that differences in the fuel-bed composition and fuel moisture content can have on the climate impact of the BBAs produced. The calculated RFE_663 nm_ values from the single-fuel measurements of Pokhrel et al.^32^ and McClure et al.^34^ span even wider ranges (−0.022 to +0.050 W/m^2^ and −0.022 to +0.022 W/m^2^, respectively), implying significant differences in the interpretation of these BBAs for radiative forcing. Specifically, the much lower SSA_663 nm_ values possible from individual fuels, compared to those from the present study, translate into greater RFE_663 nm_ values and a larger inferred contribution to warming (or at least less cooling) than our measurements would suggest. These differences are most pronounced at correspondingly low values of AAE (<1.5) that imply that absorption is dominated by the BC component. For such BBAs, then, the mixed fuel beds of the present study lead to a higher SSA_663 nm_, thus implying a larger relative amount of scattering, than was found in the single-fuel studies. This observation makes sense since the fuel beds contain more components that are likely to smolder and thereby contribute to the production of organic particulate matter, which increases light scattering but only contributes to light absorption slightly (if at all). Taken together, these comparisons suggest that it is vital to account for fuel-bed composition and moisture content to represent the climate impacts of BBAs from wildland fires accurately.

## Data Availability

The data underlying this study are openly available in Figshare at https://doi.org/10.6084/m9.figshare.c.7368346.

## Supporting Information Available

Table of the intensive optical properties for each burn and a table listing the treatment of literature data presented here. Figures include a photograph of an example fuel bed, a map of the Southeastern United States with eco-regions studied indicated, a plot of AAE and SSA vs the modified combustion efficiency, and a plot of the radiative forcing efficiency vs AAE (PDF)
