# Supplementary material for: Optical Properties of Biomass Burning Aerosols from Simulated Wildfires and Prescribed Fires with Representative Fuel Beds from the Southeast United States
Source: ACS EST Air. 2024 Aug 10;1(9):1137–46. doi: 10.1021/acsestair.4c00091 (PMC11406515; doi:10.1021/acsestair.4c00091)
Supplement: Supplementary file 1 — ea4c00091_si_001.pdf [file ea4c00091_si_001.pdf]

# Supporting Information for: Optical Properties of Biomass Burning Aerosols from Simulated Wildfires and Prescribed Fires with Representative Fuel Beds from the Southeast United States

*Zachary C. McQueen [a], Ryan P. Poland [a], Chase K. Glenn [b†], Omar El Hajj [b‡], Robert Penland [b], Anita Anosike [b], Kruthika V. Kumar [b], Joseph J. O'Brien [c], Rawad Saleh [b], Geoffrey D. Smith [a]**

[a] Department of Chemistry, University of Georgia, Athens, Georgia, 30602, United States

[b] School of Environmental, Civil, Agricultural and Mechanical Engineering, University of Georgia, Athens, Georgia, 30602, United States

[c] U.S. Department of Agriculture Forest Service, Southern Research Station, Athens Prescribed Fire Science Laboratory, Athens, Georgia, 30602, United States

*Email: geosmith@uga.edu

[†] Now at Aerodyne Research, Billerica, Massachusetts, 01821, United States

[‡] Now at Tofwerk USA, Boulder, Colorado, 80301, United States

**List of Contents:**

**Table S1**: Day averaged intensive optical properties.

**Table S2:** Description of data treatment for literature comparisons.

**Figure S1**: Map of the Southeast United States and the eco-regions studied.

**Figure S2**: Photograph of an example fuel bed.

**Figure S3**: SSA and AAE plotted vs. the modified combustion efficiency.

**Figure S4**: Radiative forcing efficiency for the current study and literature comparison studies.

**Table S1: Day averaged intensive optical properties.**

| Date | Eco-Region | Condition | AAE | $AAE_{BG}$ (406 nm/532 nm) | $AAE_{RIR}$ (663 nm/783 nm) | SSA 663 nm |
|---|---|---|---|---|---|---|
| 25-Oct-22 | Piedmont | Wildfire | 1.690 | 1.863 | 0.879 | 0.832 |
| 27-Oct-22 | Piedmont | Wildfire | 1.825 | 1.979 | 1.063 | 0.859 |
| 28-Oct-22 | Piedmont | Prescribed | 3.206 | 3.525 | 1.724 | 0.959 |
| 31-Oct-22 | Piedmont | Prescribed | 2.983 | 3.300 | 1.578 | 0.949 |
| 1-Nov-22 | Piedmont | Wildfire | 1.902 | 2.108 | 1.078 | 0.874 |
| 2-Nov-22 | Coastal Plain | Wildfire | 1.536 | 1.644 | 0.892 | 0.824 |
| 3-Nov-22 | Coastal Plain | Prescribed | 2.430 | 2.802 | 1.120 | 0.927 |
| 4-Nov-22 | Coastal Plain | Wildfire | 1.324 | 1.082 | 0.685 | 0.745 |
| 7-Nov-22 | Coastal Plain | Prescribed | 2.880 | 3.261 | 1.187 | 0.937 |
| 8-Nov-22 | Coastal Plain | Wildfire | 1.425 | 1.518 | 0.834 | 0.801 |
| 9-Nov-22 | Blue Ridge | Wildfire | 3.893 | 4.366 | 1.708 | 0.984 |
| 10-Nov-22 | Blue Ridge | Prescribed | 2.422 | 2.575 | 1.521 | 0.890 |
| 11-Nov-22 | Blue Ridge | Prescribed | 2.974 | 3.072 | 2.013 | 0.938 |
| 12-Nov-22 | Blue Ridge | Wildfire | 3.511 | 3.852 | 1.634 | 0.977 |
| 14-Nov-22 | Blue Ridge | Wildfire | 3.805 | 4.272 | 1.593 | 0.986 |
| 15-Nov-22 | Blue Ridge | Prescribed | 2.596 | 2.820 | 1.552 | 0.933 |

**Table S2: Description of data treatment for literature comparisons.**

| Study | Instruments | wavelengths (nm) | AAE plotted in Figure 5 | $SSA_{663nm}$ plotted in Figure 5 |
|---|---|---|---|---|
| This study | MultiPAS-IV[1]; CRDS | Absorption: 406, 532, 663, 783<br>Extinction: 663 | AAE is derived from a power law fit to all four absorption coefficients. | SSA is calculated from the absorption and extinction coefficients at 663 nm. |
| Pokhrel et al.[2] FLAME-IV | PAS (Lack 2012); CRDS (Langridge 2011) | Absorption/Extinction: 405, 532, 660 | AAE values are taken as reported by Pokhrel et al., as calculated from the slope of a linear fit to the log of the absorption vs the log of wavelength using all three wavelengths. | SSA at 663 nm is calculated from values of absorption and extinction at 663 nm, which are extrapolated using the AAE and EAE values reported by Pokhrel et al. |
| McClure et al.[3,4] FIREX | PASS-3 (DMT) (781 nm); CRD-PAS (406 nm, 532 nm) | Absorption/Extinction: 405, 532, 781 | AAE values are taken as reported by McClure et al. as calculated from absorption values at 405 nm and 532 nm. | SSA at 663 nm was calculated from absorption and extinction at 663 nm, which were derived by extrapolation using the dual-wavelength (405 nm and 532 nm) AAE and EAE values reported by McClure et al. |
| Marsavin et al.[5] | CLAP (NOAA/GMD); Integrating Nephelometer (TSI) | Absorption: 467 528, 652<br>Scattering: 450, 550, 700 | AAE values are taken as reported by Marsavin et al. as calculated from absorption measured at 467 nm and 652 nm. | SSA at 663 nm was calculated from absorption and scattering at 663 nm, which were derived by extrapolation using the values of AAE (467 nm and 652 nm) and SAE (450 nm and 550 nm) reported by Marsavin et al. |

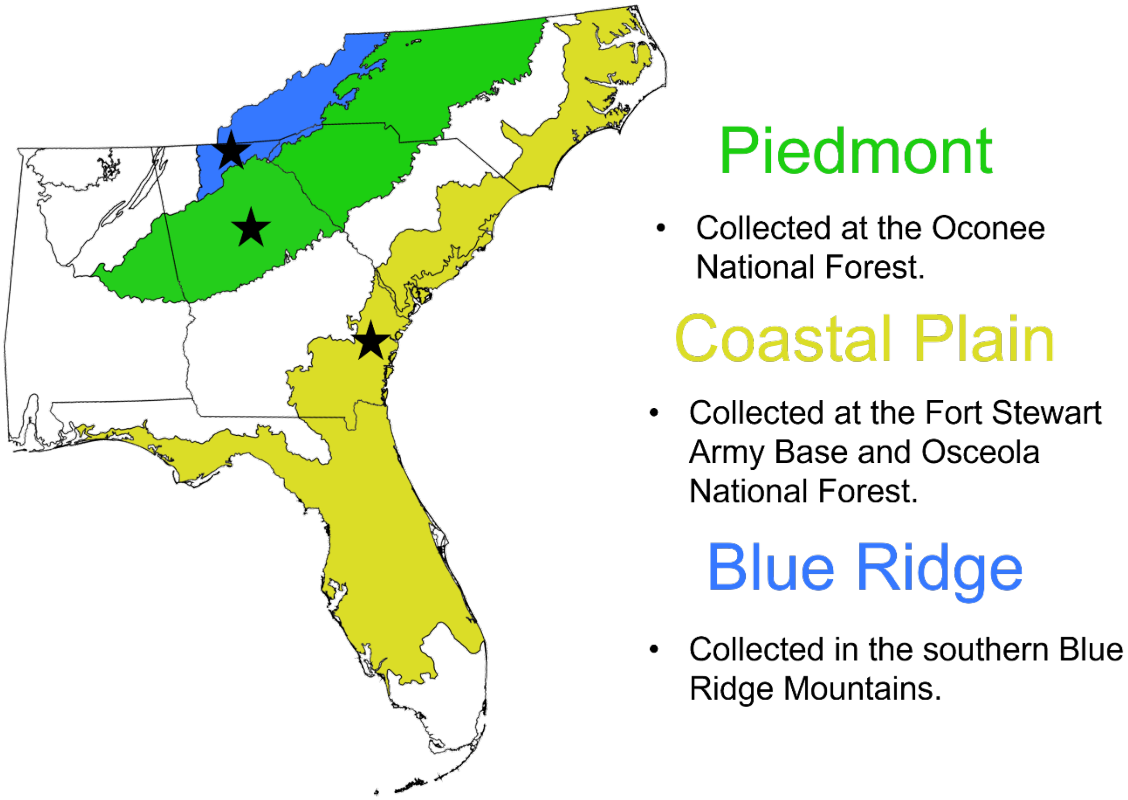


**Figure S1: Map of the Southeast United States color coded by the eco-regions studied in this work. The stars represent the approximate location of fuel collection.**

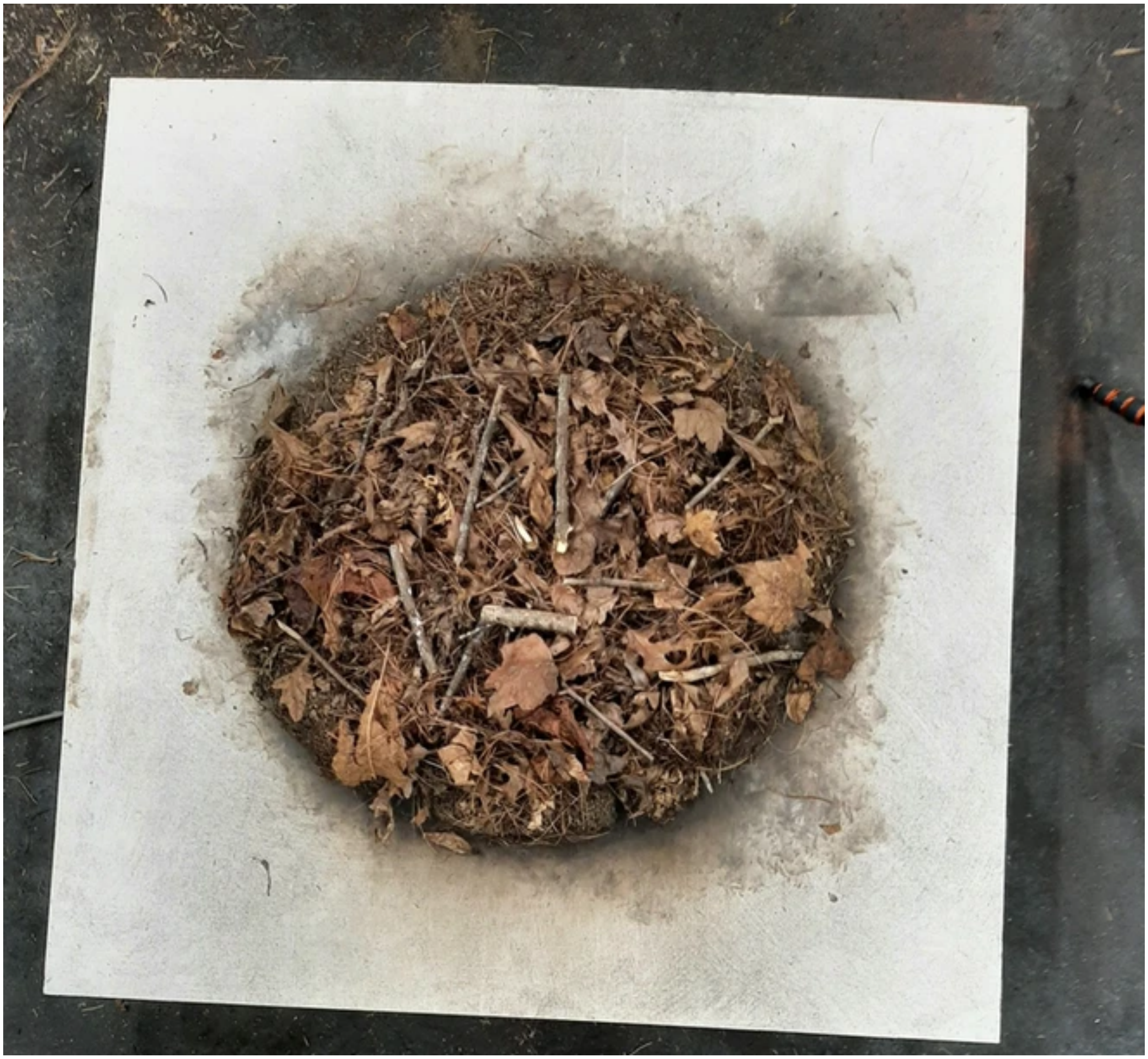

**Figure S2: Photograph of an example fuel bed. This fuel bed was from the Piedmont eco-region.**

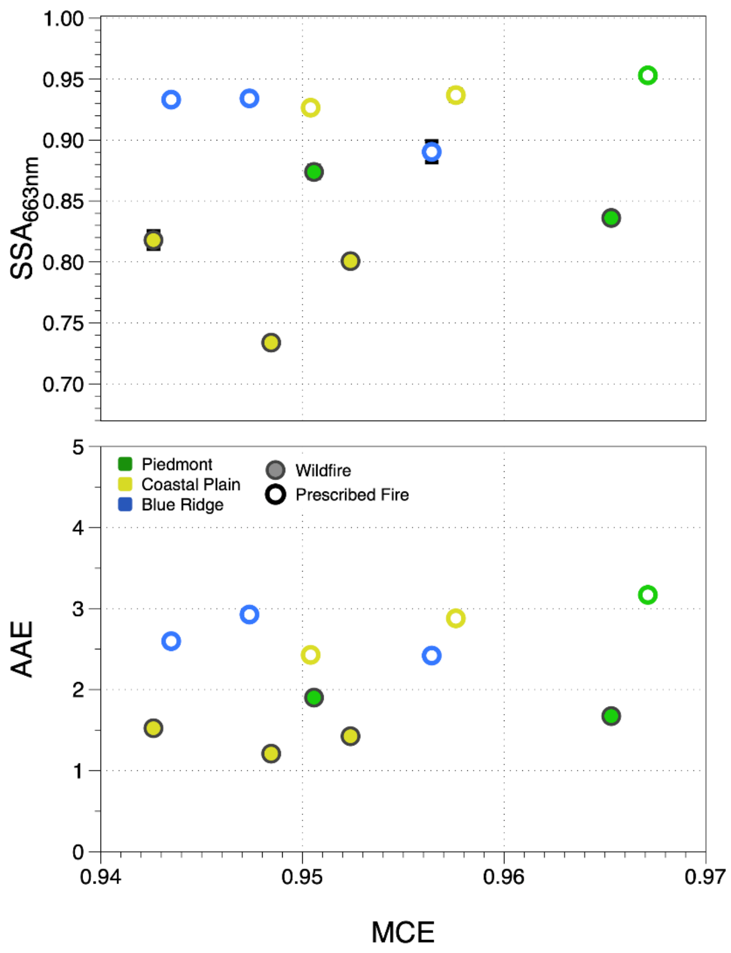


**Figure S3: The AAE and $SSA_{663}$ plotted versus the modified combustion efficiency (MCE). The MCE is defined as the ratio of $CO_2$ concentration to the sum of CO and $CO_2$ where high MCE is a more "efficient" combustion. There is a low degree of correlation observed between AAE and $SSA_{663}$ with MCE. CO emissions for Blue Ridge wildfire fuel beds were very high and saturated the CO monitor, which would underestimate the CO concentrations. For this reason, data points for BRW fuel beds were left out of the figure.**

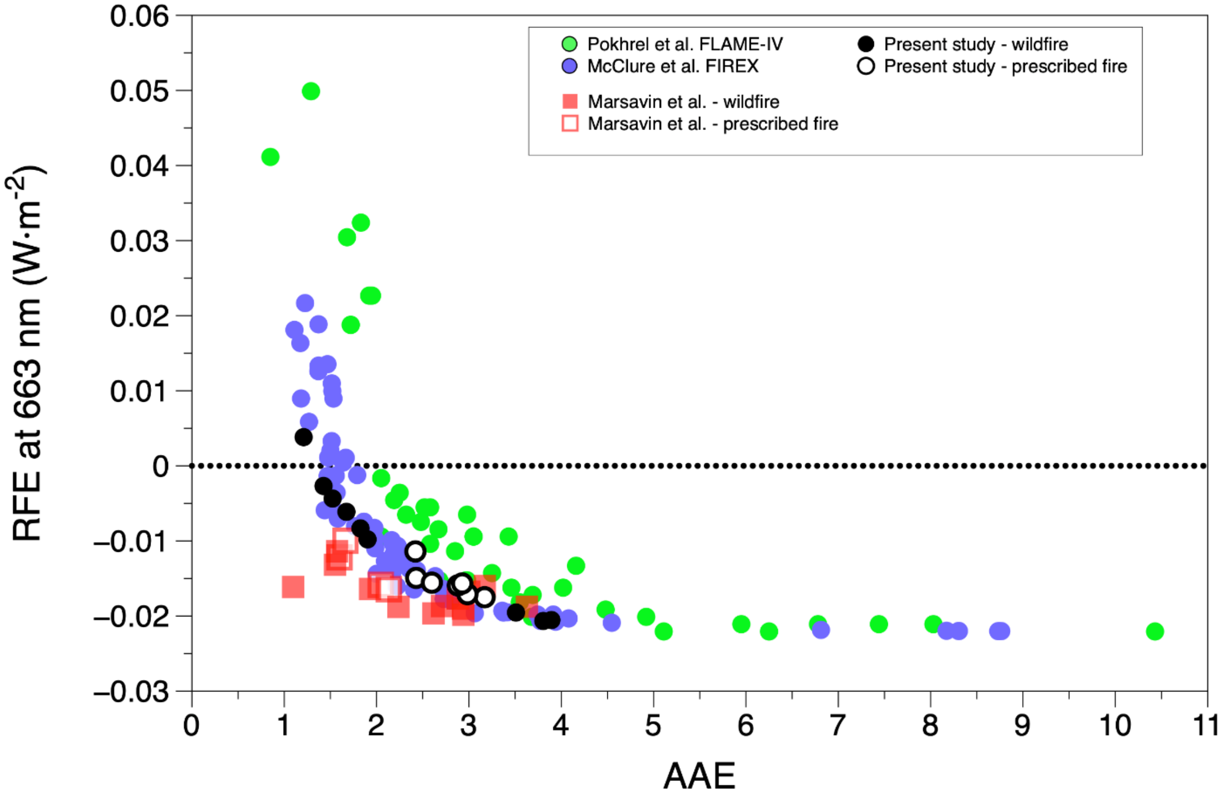


**Figure S4: Radiative forcing efficiency (RFE) at 663 nm calculated for the present study and the three literature studies used for comparison in Section 3.4 using Equation 5 in the main text.**

**References**

(1) Fischer, D. A.; Smith, G. D. A Portable, Four-Wavelength, Single-Cell Photoacoustic Spectrometer for Ambient Aerosol Absorption. *Aerosol Sci Tech* **2018**, *52* (4), 393–406. https://doi.org/10.1080/02786826.2017.1413231.

(2) Pokhrel, R. P.; Wagner, N. L.; Langridge, J. M.; Lack, D. A.; Jayarathne, T.; Stone, E. A.; Stockwell, C. E.; Yokelson, R. J.; Murphy, S. M. Parameterization of Single-Scattering Albedo (SSA) and Absorption Ångström Exponent (AAE) with EC / OC for Aerosol Emissions from Biomass Burning. *Atmos Chem Phys* **2016**, *16* (15), 9549–9561. https://doi.org/10.5194/acp-16-9549-2016.

(3) McClure, C. D.; Lim, C. Y.; Hagan, D. H.; Kroll, J. H.; Cappa, C. D. Biomass-Burning-Derived Particles from a Wide Variety of Fuels – Part 1: Properties of Primary Particles. *Atmos Chem Phys* **2020**, *20* (3), 1531–1547. https://doi.org/10.5194/acp-20-1531-2020.

(4) Cappa, C.; Lim, C.; Hagan, D.; Kroll, J. Measurements from the Fire Influence on Regional and Global Environments Experiment (FIREX) Fire Lab Mini Chamber Experiment. *Dryad Data Repository*, **2019**. https://datadryad.org/stash/dataset/doi:10.25338/B8CK5N (accessed: 2024-04-23). https://doi.org/10.25338/b8ck5n.

(5) Marsavin, A.; Gageldonk, R. van; Bernays, N.; May, N. W.; Jaffe, D. A.; Fry, J. L. Optical Properties of Biomass Burning Aerosol during the 2021 Oregon Fire Season: Comparison between Wild and Prescribed Fires. *Environ Sci Atmospheres* **2023**, *3* (3), 608–626. https://doi.org/10.1039/d2ea00118g.
